# Pharmacokinetic and Pharmacodynamic Analysis of the 3CL Protease Inhibitor Ensitrelvir in a SARS-CoV-2 Infection Mouse Model

**DOI:** 10.3390/v15102052

**Published:** 2023-10-05

**Authors:** Keita Fukao, Haruaki Nobori, Takayuki Kuroda, Kaoru Baba, Kazumi Matsumoto, Yukari Tanaka, Yuki Tachibana, Teruhisa Kato, Takao Shishido

**Affiliations:** 1 Pharmaceutical Research Division, Shionogi & Co., Ltd., 1-1, Futaba-cho 3-chome, Toyonaka 561-0825, Osaka, Japan; 2 Research Area for Drug Candidate Generation II, Shionogi TechnoAdvance Research Co., Ltd., 1-1, Futaba-cho 3-chome, Toyonaka 561-0825, Osaka, Japan

**Keywords:** antiviral, SARS-CoV-2, COVID-19, 3C-like protease, delayed-treatment mouse model

## Abstract

The small-molecule antiviral drug ensitrelvir targets the 3C-like protease of severe acute respiratory syndrome coronavirus 2 (SARS-CoV-2). This study evaluated its inhibitory effect on viral replication in a delayed-treatment mouse model and investigated the relationship between pharmacokinetic (PK) parameters and pharmacodynamic (PD) effects. SARS-CoV-2 gamma-strain-infected BALB/c mice were orally treated with various doses of ensitrelvir starting 24 h post-infection. Effectiveness was determined 48 h after first administration based on lung viral titers. Ensitrelvir PK parameters were estimated from previously reported plasma concentration data and PK/PD analyses were performed. Ensitrelvir doses ≥ 16 mg/kg once daily, ≥8 mg/kg twice daily, or ≥8 mg/kg thrice daily for two days significantly reduced lung viral titers compared to that of the vehicle. PK/PD analyses revealed that mean AUC_0–48h_ post-first administration, plasma concentration 48 h post-first administration (C_48h_), and total time above the target plasma concentration (Time_High_) were PK parameters predictive of viral titer reduction. In conclusion, ensitrelvir dose-dependently reduced lung SARS-CoV-2 titers in mice, suggesting it inhibited viral replication. PK parameters C_48h_ and Time_High_ were associated with sustained ensitrelvir plasma concentrations and correlated with the reduced viral titers. The findings suggest that maintaining ensitrelvir plasma concentration is effective for exerting antiviral activity against SARS-CoV-2.

## 1. Introduction

Coronavirus disease 2019 (COVID-19) is a global health issue caused by a novel coronavirus, severe acute respiratory syndrome coronavirus 2 (SARS-CoV-2), which is highly pathogenic. There have been over 770 million confirmed cases of COVID-19 reported worldwide and over 6.9 million deaths as of 27 August 2023 [1]. The devastating impact of SARS-CoV-2 and COVID-19 highlights the critical need for the continued identification and development of safe and effective therapeutic agents. When it comes to managing SARS-CoV-2 infection and COVID-19, it is likely that a combination of vaccination strategies and oral antiviral medications may be the most beneficial approach.

SARS-CoV-2 is a member of *Coronaviridae*, with each virion consisting of four structural proteins, including the spike (S) protein [2]. The S protein is a component of the viral membrane and through its receptor-binding domain directly interacts with receptor angiotensin-converting enzyme 2 (ACE2) present on target host cells [3,4]. While the biological properties of the S protein have made it the primary target for vaccine development against COVID-19, mutations in the S protein can lead to variants that may reduce the efficacy of existing vaccines. For instance, a variety of virus variants have emerged since the beginning of the COVID-19 pandemic with characteristic mutations accumulating in the S protein [5], including five variants of concern (VOCs) that are classified as alpha, beta, gamma, delta, and omicron variants. This variation and the risk of vaccines losing their efficacy as the viruses evolve increases the need for alternative treatment options for individuals that may become infected with SARS-CoV-2.

Several oral therapeutic agents have been considered for treating patients with COVID-19. Those approved for use or showing potential for treating SARS-CoV-2 infection target the viral RNA-dependent RNA polymerase (RdRp) or the 3C-like (3CL) protease. Clinically developed agents include molnupiravir (the prodrug form of the antiviral nucleotide analogue, β-d-N_4_-hydroxycytidine), which targets RdRp, and nirmatrelvir, which targets 3CL protease [6,7,8]. Another SARS-CoV-2 RdRp-targeting drug is remdesivir, which is a nucleotide analog prodrug originally developed to treat individuals with Ebola virus infections.

The targeting of viral proteases for oral antiviral therapies has been demonstrated to be effective in treating patients with HIV and hepatitis C virus infections [9,10]. A recent compound, ensitrelvir fumaric acid (formerly S-217622 fumaric acid, hereafter ensitrelvir), was specifically developed as a SARS-CoV-2 3CL protease inhibitor and is currently in clinical development for the treatment of COVID-19 [11,12,13,14]. Several biological characteristics of SARS-CoV-2 3CL protease make it an ideal target for antiviral therapeutics. First, two polyproteins encoded by the SARS-CoV-2 genome are cleaved at 11 different sites by 3CL protease to generate multiple non-structural proteins that are necessary for viral replication [15]. Second, the protease is conserved among coronaviruses, yet no human proteases with the same substrate specificity have been identified [16]. Ensitrelvir demonstrates in vitro efficacy against various SARS-CoV-2 variants, including different VOC strains, exhibits in vivo efficacy in mice and hamsters when administered immediately or after SARS-CoV-2 infection, and significantly reduces viral titers in nasal lavage fluid in Phase 2a and Phase 2b studies [11,12,13,14,17,18,19]. Furthermore, ensitrelvir received emergency regulatory approval from the Ministry of Health, Labour, and Welfare in Japan on 22 November 2022 as a therapeutic drug for SARS-CoV-2 infection.

Initial assessments of potential antiviral candidates are often based on in vitro potency; however, in vivo, pharmacokinetic (PK) properties, such as plasma protein binding, drug interactions, and tissue distribution, may be critical for the successful transition to effective clinical use [20]. Thus, both preclinical and clinical data are necessary for determining the optimal clinical dosing regimens for predicting and maximizing antiviral efficacy in patients [21,22,23,24]. Accordingly, PK and pharmacodynamic (PD) studies have been conducted for various antiviral agents, including those for use against influenza virus [24,25], respiratory syncytial virus [26], herpes simplex virus [27], and SARS-CoV-2 [28].

The current study was a continued evaluation of the novel antiviral agent ensitrelvir. We used a delayed-treatment mouse model system to evaluate the inhibitory effect of ensitrelvir on SARS-CoV-2 replication in the lungs of mice following a 24 h delay in treatment. The relationship between PK parameters and PD effect of ensitrelvir was also evaluated and correlated with ensitrelvir in vivo efficacy. This is the first report of PK/PD analysis of 3CL protease inhibitors using an in vivo model of SARS-CoV-2 infection. The findings from this study should not only further the evaluation of ensitrelvir efficacy, but also provide critical data for determining optimal clinical dosing regimens for this novel antiviral drug in the treatment of patients with COVID-19.

## 2. Materials and Methods

### 2.1. Ethics

The design and execution of this study were in accordance with the Declaration of Helsinki and consistent with Article 43 of the Regulation for Enforcement of the Act on Securing Quality, Efficacy, and Safety of Products Including Pharmaceuticals and Medical Devices (Standards for the Reliability of Application Data; https://www.japaneselawtranslation.go.jp/en/laws/view/3215/en, accessed on 23 August 2021). The animal protocols were reviewed by the Shionogi Pharmaceutical Research Centre Institutional Animal Care and Use Committee, Shionogi & Co., Ltd., Toyonaka, Japan (approval no: S21140D-0001 and S21068D-0015). The animal facilities were accredited by the Association for Assessment and Accreditation of Laboratory Animal Care International.

### 2.2. Cell Lines and Viruses

Monkey-kidney-derived Vero E6 cells expressing transmembrane serine protease 2 (VeroE6/TMPRSS2; RRID:CVCL_YQ49) [29] were obtained from the Japanese Collection of Research Bioresources Cell Bank (Osaka, Japan) and maintained in Dulbecco’s Modified Eagle Medium (DMEM; Thermo Fisher Scientific, Waltham, MA, USA) supplemented with 10% heat-inactivated fetal bovine serum (FBS; Sigma-Aldrich Co. Ltd., St. Louis, MO, USA) and 1% penicillin-streptomycin (Thermo Fisher Scientific) as previously described [12]. The SARS-CoV-2 gamma strain hCoV-19/Japan/TY7-501/2021 (Pango lineage P.1) was provided by the National Institute of Infectious Diseases of Japan.

### 2.3. Anti-SARS-CoV-2 Test Compound

Ensitrelvir (Shionogi & Co. Ltd., Osaka, Japan) was synthesized as previously described [11] and freshly prepared to the desired concentrations in 0.5% (*w*/*v*) Methylcellulose 400cP (MC) solution (Fujifilm Wako Pure Chemical Corp., Osaka, Japan). The indicated doses of ensitrelvir represent its free form. Ensitrelvir and MC vehicle dosing was via oral administration as described below.

### 2.4. Animals and Study Design

**Figure 1:**
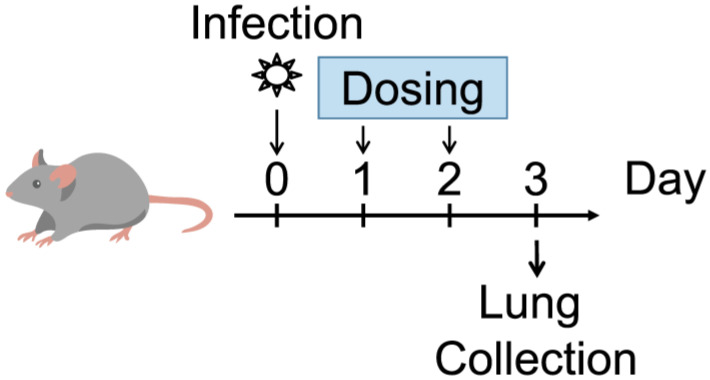
Study design. Female BALB/c mice were intranasally infected with 1.00 × 10^4^ TCID_50_ of SARS-CoV-2 gamma strain hCoV-19/Japan/TY7-501/2021. The mice were then treated with various doses of ensitrelvir or vehicle (0.5% (*w*/*v*) Methylcellulose 400cP) starting 24 h post-infection. The treatment schedule was one dose (1 d post-infection) or a dose every 24 h (once daily), every 12 h (twice daily), or every 8 h (thrice daily) for 2 d. The vehicle control group was treated twice daily for 2 d. Lungs were collected 3 d post-infection (48 h after the first treatment). N = 5 mice/group.

Female BALB/cAJcl mice were purchased from CLEA Japan, Inc. (Tokyo, Japan) and used at 5 weeks of age. The mice were maintained and treated as previously detailed [12]. The study design is summarized in Figure 1. Specifically, the mice were anesthetized by intramuscular administration of 100 μL anesthetic solution containing 0.03 mg/mL medetomidine hydrochloride (Nippon Zenyaku Kogyo Co., Ltd., Fukushima, Japan), 0.4 mg/mL midazolam (Maruishi Pharmaceutical Co., Ltd., Osaka, Japan), and 0.5 mg/mL butorphanol tartrate (Meiji Seika Pharma Co., Ltd., Tokyo, Japan) in saline, and then each mouse was intranasally inoculated with 50 μL of virus suspension containing 1.00 × 10^4^ TCID_50_ of SARS-CoV-2 gamma strain (hCoV-19/Japan/TY7-501/2021). Ensitrelvir was orally administered at 8, 16, 32, or 64 mg/kg. The treatment schedule was one shot or a shot every 24 h (once daily), every 12 h (twice daily), or every 8 h (thrice daily) for 2 d. The experimental groups treated with one shot received 32 or 64 mg/kg ensitrelvir. The experimental groups treated once daily received 16, 32, or 64 mg/kg ensitrelvir per dosing, while the groups treated twice daily received 8, 16, 32, or 64 mg/kg ensitrelvir per dosing, and the groups treated thrice daily received 8, 16, 32, or 64 mg/kg ensitrelvir per dosing. No adverse effects were observed in rodents under the same exposure conditions as the maximum dose of ensitrelvir administered to mice. The untreated (vehicle) control group received 0.5% (*w*/*v*) MC only, twice daily. Each treatment group consisted of five mice. The first administration of ensitrelvir or vehicle was performed 24 h after virus infection. At the time this study was conducted, nirmatrelvir had not been launched and could not be evaluated at the same time. We have subsequently confirmed that nirmatrelvir demonstrates antiviral activity in this model [14].

### 2.5. Viral Titer Evaluation

The SARS-CoV-2 virus was propagated and titered on VeroE6/TMPRSS2 cells as previously described [12]. Viral titers in the lungs of infected mice were evaluated 48 h after the first administration. Briefly, the mice were sacrificed under isoflurane anesthesia and lung homogenates were prepared, serially diluted with viral assay medium, and added to wells containing VeroE6/TMPRSS2 cells as previously detailed [12]. After incubating at 37 °C for 4 d, the virus-induced cytopathic effect (CPE) was evaluated using a microscope. Viral titers were expressed as log_10_ TCID_50_/mL. If no CPE was observed at the lowest dilution, the titer was defined as 1.80-log_10_ TCID_50_/mL.

### 2.6. Pharmacokinetic Simulation

The PK simulation was performed as previously described [11,24]. The PK parameters for ensitrelvir plasma concentrations were calculated using Phoenix WinNonlin Version 8.1 software (Certara, Princeton, NJ, USA). Plasma concentrations of all dosing groups in the PD study were simulated by non-parametric analysis of plasma concentration data obtained from a previous PK study [11]. The following PK parameters were calculated based on the non-compartmental method: maximum concentration (C_max_), AUC_0–48h_ post-first administration, concentration 48 h post-first administration (C_48h_), and total time above the target plasma concentration (Time_High_).

### 2.7. Pharmacokinetic and Pharmacodynamic Analysis

A nonlinear regression analytical model was applied to the PD data and PK parameters of the test substance groups using XLfit, Version 5.3.1.3 software (ID Business Solutions, Woking, UK) [24]. These data were fitted to a four-parameter logistic equation (model 205):

y = A + ((B − A)/(1 + ((C/x)^D^)))

where y is the difference of the log_10_ TCID_50_/mL between each dosing group and the vehicle group; A is minimum y (locked at −4.77), which is the difference between the lower limit of quantification of viral titer, i.e., 1.80-log_10_ TCID_50_/mL, and the mean value of vehicle viral titer, i.e., 6.57-log_10_ TCID_50_/mL; B is maximum y (locked at 0); C is 50% efficacy concentration (EC_50_, the value of a model parameter that produced a 50% maximum effect); x is the value of each PK parameter [C_max_/protein adjusted (PA)-EC_50_, AUC_0–48h_/PA-EC_50_, C_48h_/PA-EC_50_, and Time_High_]; and D is the slope factor. The PA-EC_50_ against SARS-CoV-2 gamma strain (hCoV-19/Japan/TY7-501/2021) value extrapolated to 100% mouse serum was 3.93 µmol/L, which is equal to 2090 ng/mL [11]. The Time_High_ (10× PA-EC_50_) for 8 mg/kg twice daily for 2 d was calculated to be zero; however, as zero could not be entered into the XLfit, Version 5.3.1.3 software, this value was converted to 0.00100 for the PK/PD analysis.

### 2.8. Viral Replication Inhibition Assays

The inhibitory effect of ensitrelvir on SARS-CoV-2 replication in cultured cells in the presence of human serum was evaluated using viral replication inhibition assays [11,12,14]. Briefly, ensitrelvir was diluted with dimethyl sulfoxide and mixed with an assay medium containing various concentrations of human serum. After incubating at room temperature for approximately 1 h, VeroE6/TMPRSS2 cells adjusted to 3.0 × 10^5^/mL and 1000 TCID_50_ SARS-CoV-2 gamma strain (hCoV-19/Japan/TY7-501/2021) were added to each sample and incubated at 37 °C for 4 d. The inhibitory effects of ensitrelvir on SARS-CoV-2-induced CPE were measured by determining cell viability using a CellTiter-Glo^®^ 2.0 Assay (Promega, Madison, WI, USA). The EC_50_ was calculated in the presence of each concentration of serum using XLfit, Version 5.3.1.3 software and the PA-EC_50_ values were extrapolated to 100% serum using linear regression. The potency shift (PS) was extrapolated to 100% serum by dividing the PA-EC_50_ value of 100% human serum by the EC_50_ in the absence of human serum.

### 2.9. Statistical Analysis

All statistical analyses were performed using SAS statistical analysis software, version 9.4 (SAS Institute, Cary, NC, USA). The two-sided significance level was set at 0.05. Mean body weight uniformity of the mice among experimental groups was confirmed by one-way analysis of variance. Endpoint analysis of viral titers in the lung tissues was measured on a logarithmic scale. The results from each ensitrelvir-treated group were compared to the corresponding vehicle groups at each sampling time point to assess the effect of ensitrelvir on viral titers in the lungs. Dunnett’s method was applied to adjust for multiplicity of multiple testing.

## 3. Results

### 3.1. Effect of Ensitrelvir Treatment on SARS-CoV-2 Titers in the Lungs of Mice

**Figure 2:**
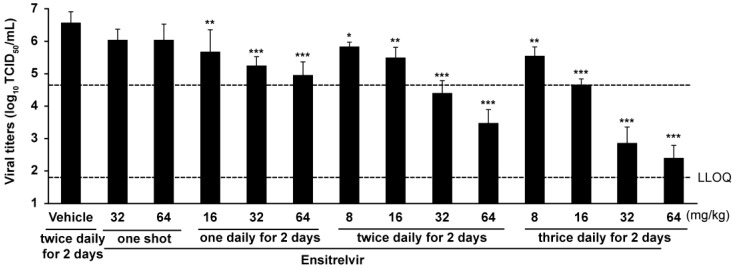
SARS-CoV-2 titers in the lungs of mice following ensitrelvir treatment. Mice were infected with 1.00 × 10^4^ TCID_50_ of SARS-CoV-2 gamma strain hCoV-19/Japan/TY7-501/2021 (Pango lineage P.1). Treatment was subsequently initiated 24 h post-infection. Treatment dosing included various concentrations of ensitrelvir (8, 16, 32, or 64 mg/kg) or vehicle (0.5% Methylcellulose 400cP, MC). The treatment schedule included one shot (single dosing), once daily (every 24 h) for 2 d, twice daily (every 12 h) for 2 d, or thrice daily (every 8 h) for 2 d. The vehicle control group received MC solution twice daily for 2 d. Viral titers were then measured in the lungs of the mice 3 d post-infection. The graph shows the mean viral titers of 5 mice/group. Error bars demonstrate standard deviations. The upper dotted line indicates the mean viral titers in the untreated group 1 d post-infection. The lower dotted line indicates 1.80-log_10_ TCID_50_/mL, the lower limit of quantification (LLOQ). The *p*-values comparing the ensitrelvir-treated groups versus the vehicle-treated group were calculated using Dunnett’s test. * *p* < 0.05, ** *p* < 0.01, *** *p* < 0.0001. The data of the twice daily for 2 d groups were previously reported [12]. SARS-CoV-2, severe acute respiratory syndrome coronavirus 2.

To evaluate the effect of delayed ensitrelvir treatment against SARS-CoV-2 gamma strain (hCoV-19/Japan/TY7-501/2021) infection, mice were orally administered ensitrelvir at various doses 24 h after infection, and the viral titers were measured at 72 h post-infection, which was 48 h after the first administration of ensitrelvir. As shown in Figure 2, ensitrelvir reduced lung viral titers compared to vehicle alone and did so in a dose-dependent manner. Specifically, the mean viral titer 72 h post-infection was significantly lower in all the ensitrelvir treatment groups compared to that of the vehicle treatment group, except for those that received a single day of treatment (*p* = 0.0065, 16 mg/kg once daily; *p* < 0.0001, 32 and 64 mg/kg once daily; *p* = 0.0423, 8 mg/kg twice daily; *p* = 0.0007, 16 mg/kg twice daily; *p* < 0.0001, 32 and 64 mg/kg twice daily; *p* = 0.0015, 8 mg/kg thrice daily; *p* < 0.0001, 16, 32, and 64 mg/kg thrice daily). The data for 2 d of twice daily ensitrelvir treatment has previously been reported [12]. The raw data used in Figure 1 are available in Table S1. As shown in Figure S1, even when treatment was delayed 72 h post-infection, ensitrelvir administered twice daily for 2 d at ≥ 8 mg/kg significantly lowered lung viral titers compared to the vehicle (8 mg/kg, *p* < 0.001; 16, 32, and 64 mg/kg, *p* < 0.0001 each).

### 3.2. PK Simulation of Ensitrelvir in SARS-CoV-2-Infected Mice

The profiles of ensitrelvir plasma concentrations in infected mice were simulated using data from a previous report [11]. The calculated PK parameters C_max_, AUC_0–48h_, and C_48h_ are presented in Table 1. The simulation included the findings for single dosing (one shot) and repeated dosing (once daily for 2 d, twice daily for 2 d, and thrice daily for 2 d). The findings for the 2 d treatments are graphically shown in Figure S2.

### 3.3. PK/PD Analysis of Ensitrelvir in SARS-CoV-2-Infected Mice

**Figure 3:**
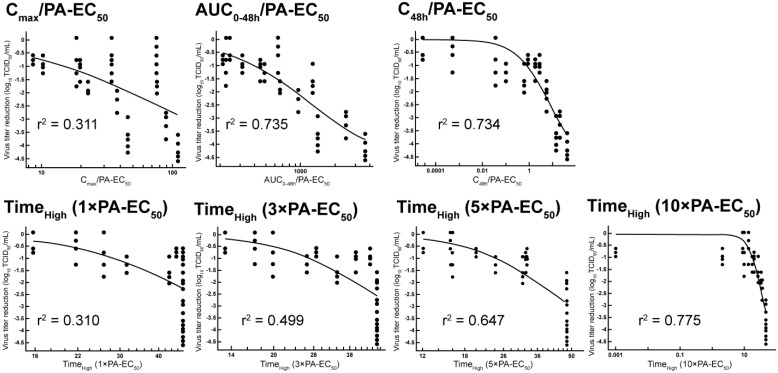
Pharmacokinetic (PK) and pharmacodynamic (PD) analysis of ensitrelvir of SARS-CoV-2-infected mice. The PK parameters maximum concentration (C_max_), AUC_0–48h_ post-first administration, concentration at 48 h post-first administration (C_48h_), and total time above the target plasma concentration (Time_High_) were calculated using the non-compartmental method. The parameters relative to protein-adjusted 50% efficacy concentration (PA-EC_50_) are plotted versus the virus titer reduction in the graphs above. Fitting curves of PD data and PK parameters using a nonlinear regression model are depicted. The coefficients of determination (r^2^) are indicated. The PA-EC_50_ value was based on a previous report [12]. AUC_0–48h_, area under the curve plasma concentrations 0 through 48 h post-first administration; SARS-CoV-2, severe acute respiratory syndrome coronavirus 2.

To investigate the relationship between the antiviral activity of ensitrelvir and PK parameters, PK/PD analyses were conducted using a nonlinear regression model. The estimated values of the PK parameters for ensitrelvir in the plasma of SARS-CoV-2-infected mice following oral treatment are presented in Table 2. The findings include single dosing (one shot) and repeated dosing (once daily for 2 d, twice daily for 2 d, and thrice daily for 2 d). Parameters of C_max_ for PA-EC_50_, AUC_0–48h_ for PA-EC_50_, C_48h_ for PA-EC_50_, and Time_High_ were estimated as the time for ensitrelvir levels to continue exceeding 1×, 3×, 5×, and 10× the PA-EC_50_ levels (1×, 3×, 5×, and 10× PA-EC_50_). The results from the four-parameter logistic equation (model 205) that was used to calculate the relationship between the PD data of viral titer reduction (log_10_ TCID_50_/mL) and PK parameters, and between PD data and Time_High_, are shown in Figure 3 and Table 3. The values of parameters A and B in the model equation for PK/PD analysis were fixed to the maximum (−4.77) and minimum (0), respectively, for the evaluation of virus titer reduction in this study. The coefficients of determination (r^2^) of Time_High_ (10× PA-EC_50_), AUC_0–48h_/PA-EC_50_, C_48h_/PA-EC_50_, Time_High_ (5× PA-EC_50_), Time_High_ (3× PA-EC_50_), C_max_/PA-EC_50_, and Time_High_ (1× PA-EC_50_) against viral titer reduction were 0.775, 0.735, 0.734, 0.647, 0.499, 0.311, and 0.310, respectively. Time_High_ (10× PA-EC_50_), AUC_0–48h_/PA-EC_50_, and C_48h_/PA-EC_50_ demonstrated coefficients of determination greater than 0.7. Both Time_High_ (10× PA-EC_50_) and C_48h_/PA-EC_50_ parameters indicated a good correlation with the PD data, suggesting that maintaining a certain level of plasma concentration is important for exerting an antiviral effect in mice infected with SARS-CoV-2 gamma strain (hCoV-19/Japan/TY7-501/2021) followed by the administration with ensitrelvir 24 h after virus infection.

### 3.4. Estimated C_48h_ Required to Achieve Certain Levels of Viral Titer Reduction

Using this SARS-CoV-2-infected mouse model and a mouse PA-EC_50_ value against SARS-CoV-2 gamma stain (hCoV-19/Japan/TY7-501/2021) of 2090 ng/mL (3.93 μmol/L) [11], the C_48h_ required to reduce viral titers by 1-log_10_, 2-log_10_, and 3-log_10_ TCID_50_/mL compared to the mean viral titer of the vehicle treatment group 48 h after the first administration of ensitrelvir was calculated to be 1610, 7900, and 30,700 ng/mL, respectively (Table 4). Similarly, using a human PA-EC_50_ value of 1610 ng/mL (3.02 μmol/L), the C_48h_ required to reduce viral titers compared to the vehicle treatment group by 1-log_10_, 2-log_10_, and 3-log_10_ TCID_50_/mL in humans was calculated to be 1240, 6090, and 23,700 ng/mL, respectively.

## 4. Discussion

As COVID-19 continues to be a major health concern worldwide, there is an urgent need for oral SARS-CoV-2-specific therapeutics to help combat infections and prevent severe disease, hospitalizations, and COVID-19-related mortality. Transitioning an antiviral drug into clinical use requires substantial effort to determine and maximize its efficacy in patients. As noted by Venisse and colleagues [20], in vivo PK properties may be critical for this, as predicted in vivo PD properties often differ from those of in vitro potency. Accordingly, both preclinical and clinical data may be essential for optimizing clinical dosing regimens [24].

In the current study, mice infected with SARS-CoV-2 gamma strain (hCoV-19/Japan/TY7-501/2021) were administered ensitrelvir 24 h after infection and its inhibitory effect on viral replication at 48 h post-first administration was evaluated. Ensitrelvir dose-dependently reduced viral titers in the lungs of mice, suggesting it was able to inhibit viral replication at the anatomically significant site of infection. Furthermore, the relationship between PK parameters and PD effects of ensitrelvir was investigated 48 h after first administration of the drug. The PK parameters Time_High_, AUC_0–48h_, and C_48h_ were found to be predictive of viral titer reduction 48 h after the first administration of ensitrelvir. Consistent with PK and PD studies that have been performed using various antiviral agents [24,25,26,27,28], we performed PK and PD analyses of ensitrelvir based on an in vivo infection model and correlated the findings with in vivo antiviral efficacy. Ensitrelvir dose-dependently reduced viral titers in the lungs of mice in the delayed-treatment model at various doses and regimens. The PK parameters C_48h_ and Time_High_ were associated with sustained ensitrelvir plasma concentrations in mice and correlated with the reduction in lung viral titers. This suggests that maintaining ensitrelvir plasma concentrations was effective for exerting its antiviral activity throughout the drug administration period. Using C_48h_ as an index, we calculated the C_48h_ required for 1-log_10_, 2-log_10_, and 3-log_10_ TCID_50_/mL virus reduction in humans to be 1240, 6090, and 23,700 ng/mL, respectively. These findings provide insight into potential dosing requirements for the clinical application of ensitrelvir for treating patients with COVID-19.

Since the administration of antivirals in clinical practice may be initiated at various times post-infection, drug efficacy is important not only during the viral growth phase, but also during the viral clearance phase. Even when the administration of ensitrelvir was delayed 72 h post-infection and given during the viral clearance phase, it demonstrated dose-dependent antiviral activity. Consistent with the outcomes observed when ensitrelvir administration was delayed 24 h post-infection, when treatment was delayed 72 h, equivalent ensitrelvir doses (≥8 mg/kg) administered twice daily for 2 d were effective in suppressing virus replication. Accordingly, using the PK/PD analysis of the 24 h delayed administration model, the estimated C_48h_ required to achieve certain levels of viral titer reduction is expected to be approximately the same, regardless of the timing of the ensitrelvir administration.

The current study had limitations. First, only a single SARS-CoV-2 strain was evaluated. However, previous studies have demonstrated efficacy of ensitrelvir against a variety of SARS-CoV-2 strains, including strains representing the five classes of VOCs [11,12,14]. Second, although small-animal models that mimic SARS-CoV-2 infection have been well-established and are essential for investigating COVID-19 and investigating potential therapeutic agents [12,14,15,30,31], obvious differences exist between humans and rodents. That noted, our current findings provide additional support for the use of small animal models for performing SARS-CoV-2 replication and infection studies. While ensitrelvir has shown antiviral activity in clinical trials [17,18], it will be important to compare our PK/PD analysis in mice to that in humans. Third, the PK parameters were simulated values, and the site of PK and PK/PD analysis was plasma instead of in the lungs, the relevant site of infection. However, the PK and PK/PD results were based on in vivo samples and correlated back to efficacy against viral infection in the lungs of the model animals. Fourth, animal studies, including ours, typically use healthy subjects without underlying health conditions. However, while aged mice infected with a mouse-adapted strain are more susceptible to severe disease, the antiviral activity of ensitrelvir is comparable to that in younger mice infected with various SARS-CoV-2 strains [14].

## 5. Conclusions

Ensitrelvir dose-dependently reduced SARS-CoV-2 titers in the lungs of mice in the delayed-treatment model at a variety of doses and regimens. The PK parameters Time_High_, AUC_0–48h_, and C_48h_ were predictive of reduced lung viral titers 48 h after the first administration of ensitrelvir in mice infected with the SARS-CoV-2 gamma strain (hCoV-19/Japan/TY7-501/2021) when ensitrelvir was administered 24 h after virus infection. This is the first report showing a correlation between C_48h_ or Time_High_, which are related to the maintenance of plasma compound concentrations, and antiviral activity of the 3CL protease inhibitor ensitrelvir in a mouse SARS-CoV-2 infection model. It is often reported that maintenance of plasma concentration is important for the efficacy of antiviral agents, but few reports have demonstrated it using in vivo models. The PK parameter(s) necessary for each mechanism of action to exert its efficacy should be determined. Relative to the development of well-tolerated and effective antiviral therapies being essential in combatting the global health threat of COVID-19, the current findings suggest that ensitrelvir has the potential to suppress viral replication and prevent clinical disease caused by SARS-CoV-2. Furthermore, PK and PK/PD analyses using the delayed-treatment mouse model may be useful for predicting clinical outcomes following the administration of ensitrelvir to patients infected with SARS-CoV-2. The results from this study clarify the plasma concentration required to demonstrate inhibition of viral replication in a delayed-treatment model of ensitrelvir. It will be important in the future to also determine the plasma concentrations required to exhibit protection against SARS-CoV-2 infection and to prevent the development of COVID-19.

## 6. Patents

Shionogi & Co., Ltd. has applied for a patent on ensitrelvir.

## Figures and Tables

**Table 1:** Pharmacokinetic parameter values of ensitrelvir in the plasma of SARS-CoV-2-infected mice.

Dosing Schedule		PK Parameters		
	Dose (mg/kg)	C_max_ (ng/mL)	AUC_0–48h_ (ng·hr/mL)	C_48h_ (ng/mL)
one shot	32	72,400	510,000	0.0591
	64	159,000	1,340,000	1.11
once daily for 2 d	16	38,800	475,000	75.8
	32	72,500	1,020,000	210
	64	160,000	2,670,000	1290
twice daily for 2 d	8	18,100	446,000	1840
	16	41,300	941,000	3760
	32	79,700	2,010,000	11,400
	64	188,000	5,230,000	42,000
thrice daily for 2 d	8	21,500	657,000	5740
	16	47,600	1,390,000	11,600
	32	96,000	2,960,000	30,000
	64	232,000	7,650,000	88,300

SARS-CoV-2, severe acute respiratory syndrome coronavirus 2; PK, pharmacokinetic; C_max_, maximum concentration; AUC_0–48h_, area under the curve plasma concentrations 0 through 48 h post-first administration; C_48h_, plasma concentration at 48 h post-first administration; one shot, single dosing; once daily, every 24 h; twice daily, every 12 h; thrice daily, every 8 h.

**Table 2:** Pharmacokinetic analysis of ensitrelvir in SARS-CoV-2-infected mice.

Dosing Schedule	Dose (mg/kg)	PK Parameters						
		C_max_/PA-EC_50_	AUC_0–48h_/PA-EC_50_	C_48h_/PA-EC_50_	Time_High_ 1× PA-EC_50_ (h)	Time_High_ 3× PA-EC_50_ (h)	Time_High_ 5× PA-EC_50_ (h)	Time_High_ 10× PA-EC_50_ (h)
one shot	32	34.6	244	0.0000283	15.8	13.3	12.1	9.09
	64	76.1	641	0.000531	21.7	17.1	15.7	13.8
once daily for 2 d	16	18.6	227	0.0363	26.7	19.9	16.0	10.1
	32	34.7	488	0.100	31.6	26.5	24.2	18.2
	64	76.6	1280	0.617	43.4	34.2	31.4	27.5
twice daily for 2 d	8	8.66	213	0.880	45.7	28.7	20.1	0.00
	16	19.8	450	1.80	48.0	40.1	32.3	20.6
	32	38.1	962	5.45	48.0	47.9	47.9	36.9
	64	90.0	2500	20.1	48.0	48.0	48.0	47.9
thrice daily for 2 d	8	10.3	314	2.75	47.9	45.2	32.7	2.13
	16	22.8	665	5.55	48.0	47.9	47.9	33.5
	32	45.9	1420	14.4	48.0	47.9	47.9	47.8
	64	111	3660	42.2	48.0	48.0	48.0	47.9

SARS-CoV-2, severe acute respiratory syndrome coronavirus 2; PK, pharmacokinetic; C_max_, maximum concentration; PA-EC_50_, protein-adjusted 50% efficacy concentration; AUC_0–48h_, area under the curve plasma concentrations 0 through 48 h post-first administration; C_48h_, plasma concentration at 48 h post-first administration; Time_High,_ total time above the target plasma concentration_;_ one shot, single dosing; once daily, every 24 h; twice daily, every 12 h; thrice daily, every 8 h.

**Table 3:** Logistic regression equation model results of pharmacokinetic parameters.

PK Parameter	Model Parameter	Estimate	r^2^
C_max_/PA-EC_50_	A	−4.77	0.311
	B	0	
	C	70.1	
	D	−0.859	
AUC_0–48h_/PA-EC_50_	A	−4.77	0.735
	B	0	
	C	1180	
	D	−1.22	
C_48h_/PA-EC_50_	A	−4.77	0.734
	B	0	
	C	6.35	
	D	−0.629	
Time_High_ (1× PA-EC_50_) (h)	A	−4.77	0.310
	B	0	
	C	50.6	
	D	−2.34	
Time_High_ (3× PA-EC_50_) (h)	A	−4.77	0.499
	B	0	
	C	44.9	
	D	−2.60	
Time_High_ (5× PA-EC_50_) (h)	A	−4.77	0.647
	B	0	
	C	40.8	
	D	−2.55	
Time_High_ (10× PA-EC_50_) (h)	A	−4.77	0.775
	B	0	
	C	33.3	
	D	−2.22	

A, maximum y (locked at −4.77); B, maximum y (locked at 0); C, EC_50_; D, slope factor. PK, pharmacokinetic; PD, pharmacodynamic; C_max_, maximum concentration; PA-EC_50_, protein-adjusted 50% efficacy concentration; AUC_0–48h_, area under the curve plasma concentrations 0 through 48 h post-first administration; C_48h_, plasma concentration at 48 h post-first administration; Time_High,_ total time above the target plasma concentration_;_ r^2^, coefficient of determination.

**Table 4:** The C_48h_ required to reduce viral titers by 1-log_10_, 2-log_10_, and 3-log_10_ TCID_50_/mL in the presence of mouse or human serum.

Viral Titer Reduction (TCID_50_/mL)	C_48h_/PA-EC_50_	Mouse PA-EC_50_ (ng/mL)	Mouse C_48h_ (ng/mL)	Human PA-EC_50_ (ng/mL)	Human C_48h_ (ng/mL)
1-log_10_	0.769	2090	1610	1610	1240
2-log_10_	3.78	2090	7900	1610	6090
3-log_10_	14.7	2090	30,700	1610	23,700

C_48h_, plasma concentration at 48 h post-first administration; PA-EC_50_, protein-adjusted 50% efficacy concentration.

## Data Availability

The data presented in this study are available from the corresponding author upon reasonable request.

## Supplementary Materials

The following supporting information can be downloaded at: https://www.mdpi.com/article/10.3390/v15102052/s1, Supplementary Methods: Mouse lung viral titers when ensitrelvir treatment was initiated 72 h post-infection; Table S1: Raw data of SARS-CoV-2 titers in the lungs of mice following ensitrelvir treatment in a 24 h delayed-treatment model (raw data of Figure 1); Figure S1: Mouse lung viral titers when ensitrelvir treatment is initiated 72 h post-infection; Supplementary Figure S2: Simulated PK parameters of ensitrelvir in the plasma of SARS-CoV-2-infected BALB/c mice.
