# Supplementary material for: Pharmacokinetic and Pharmacodynamic Analysis of the 3CL Protease Inhibitor Ensitrelvir in a SARS-CoV-2 Infection Mouse Model
Source: Viruses. 2023 Oct 5;15(10):2052. doi: 10.3390/v15102052 (PMC10612060; doi:10.3390/v15102052)
Supplement: Supplementary file 1 [file viruses-15-02052-s001.zip › viruses-2546709-supplementary.pdf]

Supplementary Materials

Supplementary Methods

Mouse lung viral titers when ensitrelvir treatment was initiated 72-h post-infection

Female BALB/cAJcl mice were purchased from CLEA Japan, Inc. (Tokyo, Japan) and used at 5 weeks of age. The mice were maintained and treated as previously detailed [12]. The mice were intranasally inoculated with 1.00×10^4 $TCID_{50}$ virus suspension (50 µL/mouse) under anesthesia. Ensitrelvir was orally dosed at 4, 8, 16, 32, or 64 mg/kg. The treatment schedule was every 12 h (twice daily) for 2 d. The untreated (Vehicle) control group received only 0.5% (w/v) MC. Each treatment group consisted of five mice. The first administration of ensitrelvir or vehicle was performed 72 h after virus infection. Viral titers in the lungs of infected mice were evaluated 120 h post-infection.

Supplementary Data

Table S1. Raw data of SARS-CoV-2 titers in the lungs of mice following ensitrelvir treatment in a 24 h delayed treatment model (raw data of Figure 1).

**Treatment**	**Dosing Schedule**	**Ensitrelvir Dose (mg/kg)**	**Viral titer (log_{10} $TCID_{50}$/mL)**									
			**Day 1**					**Day 2**				
			**1**	**2**	**3**	**4**	**5**	**1**	**2**	**3**	**4**	**5**
Vehicle	twice daily for 2 d	—	5.30	4.53	4.63	4.97	3.80	6.63	6.97	6.30	6.13	6.80
Ensitrelvir	one shot	32	—	—	—	—	—	5.97	5.80	5.80	5.97	6.63

	64	—	—	—	—	—	6.63	5.97	5.30	5.97	6.30
once daily for 2 d	16	—	—	—	—	—	6.63	5.80	5.30	5.80	4.80
	32	—	—	—	—	—	4.97	5.63	4.97	5.30	5.30
	64	—	—	—	—	—	4.80	4.97	4.80	4.53	5.63
twice daily for 2 d	8	—	—	—	—	—	5.80	5.80	5.97	5.97	5.63
	16	—	—	—	—	—	5.80	5.63	5.53	5.53	4.97
	32	—	—	—	—	—	4.30	3.80	4.63	4.63	4.63
	64	—	—	—	—	—	2.80	3.80	3.63	3.30	3.80
thrice daily for 2 d	8	—	—	—	—	—	5.53	5.30	5.63	5.30	5.97
	16	—	—	—	—	—	4.53	4.63	4.97	4.63	4.53
	32	—	—	—	—	—	2.80	2.30	2.53	2.97	3.63
	64	—	—	—	—	—	2.97	2.63	2.13	2.30	1.97

SARS-CoV-2, severe acute respiratory syndrome coronavirus 2

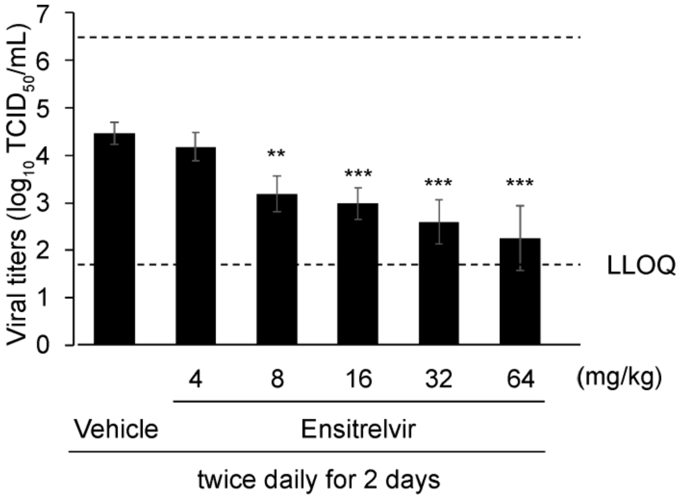

Figure S1. Mouse lung viral titers when ensitrelvir treatment is initiated 72 h post-infection. Mice were infected with 1.00 × 10^4 $TCID_{50}$ SARS-CoV-2 gamma strain hCoV-19/Japan/TY7-501/2021 (Pango lineage P.1). Treatment was then initiated 72 h post-infection. Treatment dosing included various concentrations of ensitrelvir (4, 8, 16, 32, or 64 mg/kg) or vehicle (0.5% Methylcellulose 400cP, MC). The treatment schedule included

twice daily (every 12 h) for 2 d. The vehicle control group received MC solution twice daily for 2 d. Viral titers were then measured in the lungs of the mice 5 d post-infection. The graph shows the mean titers of 5 mice/group. Error bars demonstrate standard deviations. The upper dotted line indicates the mean viral titers in the vehicle group 3 d post-infection (as per Figure 1). The lower dotted line indicates 1.80-$\log_{10}$ $TCID_{50}$/mL, the lower limit of quantification (LLOQ). The p-values comparing the ensitrelvir-treated groups versus the vehicle-treated group were calculated using Dunnett's test. **$p < 0.01$, ***$p < 0.0001$. SARS-CoV-2, severe acute respiratory syndrome coronavirus 2.

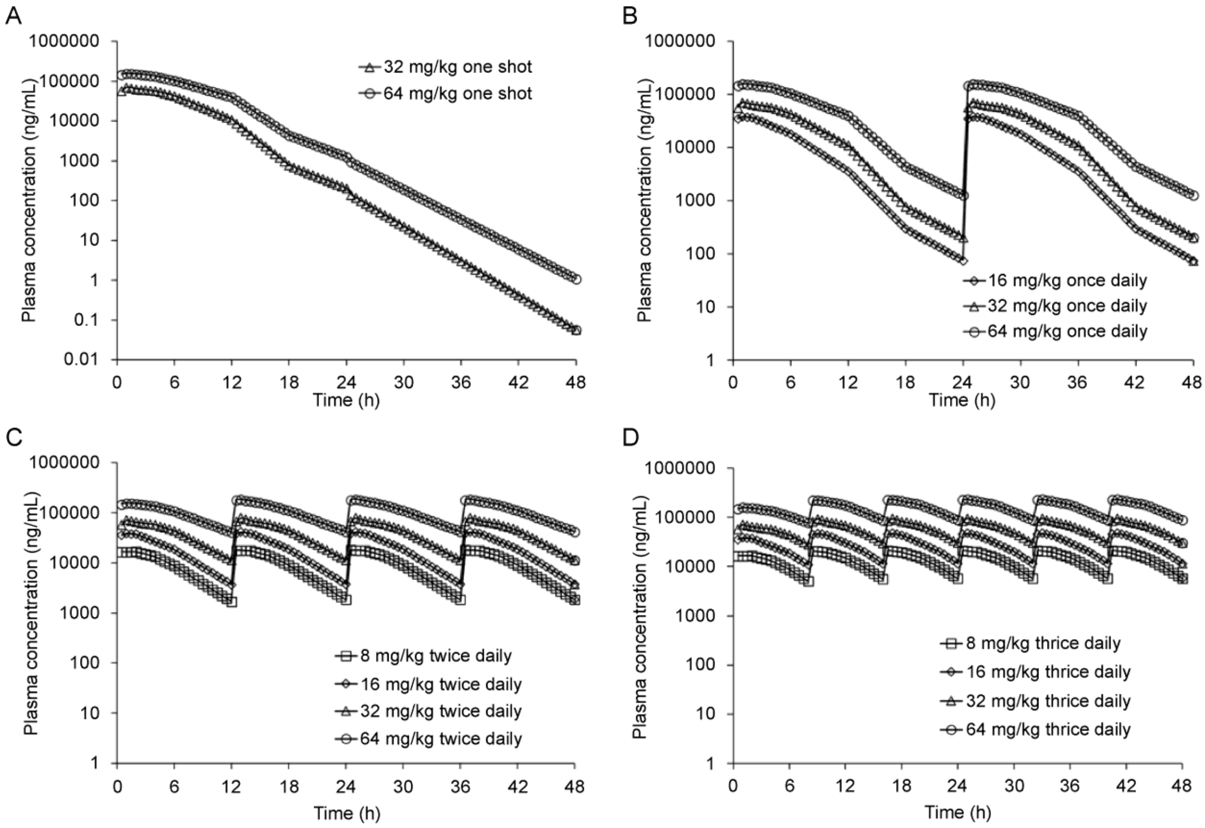

Figure S2. Estimated pharmacokinetic (PK) parameters of ensitrelvir in the plasma of SARS-CoV-2-infected BALB/c mice. The plasma concentration of ensitrelvir is plotted versus time post-first administration of 8, 16,

32, or 64 mg/kg ensitrelvir. The PK data were simulated from a previous report [12]. Each point represents the mean of 3 or 4 mice. A. Ensitrelvir dosing of one shot. B. Ensitrelvir dosing once a day (every 24 h) for 2 d. C. Ensitrelvir dosing twice a day (every 12 h) for 2 d. D. Ensitrelvir dosing thrice a day (every 8 h) for 2 d. SARS-CoV-2, severe acute respiratory syndrome coronavirus 2.
